# Exploring the implementation of an evidence-based health promotion intervention for women experiencing intimate partner violence (iHEAL) in diverse contexts: Study Protocol

**DOI:** 10.1371/journal.pone.0330285

**Published:** 2025-09-16

**Authors:** Marilyn Ford-Gilboe, Kelly Scott-Storey, Annette J. Browne, Colleen Varcoe, Caitlin Burd, Karen Campbell, Christine Garinger, Susan Jack, Kelsey Lynch, Jeannie Malcolm, Tara Mantler, Sue O’Donnell, Nancy Perrin, Jacqueline Potvin, Christina Safar, Victoria Smye, Petrea Taylor, C. Nadine Wathen

**Affiliations:** 1 Arthur Labatt Family School of Nursing, Western University, London, Ontario, Canada; 2 Faculty of Nursing, University of New Brunswick, Fredericton, New Brunswick, Canada; 3 School of Nursing, University of British Columbia, Vancouver, British Columbia, Canada; 4 School of Nursing, York University, Toronto, Ontario, Canada; 5 School of Nursing, McMaster University, Hamilton, Ontario, Canada; 6 School of Health Studies, Western University, London, Ontario, Canada; 7 School of Nursing, Johns Hopkins University, Baltimore, Maryland, United States of America; 8 Faculty of Information and Media Studies, Western University, London, Ontario, Canada; PLOS: Public Library of Science, UNITED KINGDOM OF GREAT BRITAIN AND NORTHERN IRELAND

## Abstract

**Objectives:**

This participatory, mixed methods study will explore how iHEAL, a woman-led, nurse-delivered health promotion intervention for women who have experienced intimate partner violence (IPV), can be implemented in real-world, community-based health care settings located in 3 Canadian provinces. Grounded in the Active Implementation Frameworks, the study’s primary aim is to identify the processes, resources and supports necessary to implement and sustain this novel program with fidelity while maintaining its benefits for women.

**Methods/Design:**

Over 2.5 years, each organization will plan for and deliver the iHEAL program, supported by an iHEAL Consultant. To explore implementation processes and fidelity, successes and challenges, and any value-added of iHEAL to organizations and/or communities, qualitative interviews will be conducted with 3 groups of participants: 1) organizational leaders; 2) implementation/delivery team members (nurses and supervisors); and 3) external stakeholders or agencies supporting iHEAL through referrals or other collaboration. High level notes capturing key issues and decisions at planning meetings will supplement these data. Administrative program data will be collected to assess program reach, participant engagement, and aspects of fidelity. Women participating in iHEAL will also be invited to complete pre/post intervention surveys to assess changes in key outcomes, with a subsample of 60 women to be interviewed about their experiences of iHEAL and suggestions for strengthening the program. Qualitative data will be analyzed using Rapid Team Based Qualitative Analysis and Reflective Thematic Analysis. Quantitative data will be summarized using descriptive statistics; pre-post intervention changes in outcomes collected in women’s surveys will be analyzed using paired t-tests. Ethical approval has been obtained, and all participants will provide informed consent.

**Significance:**

The findings of this research are expected to yield insights about organizational factors that shape the delivery of iHEAL and support the development of guidance materials for future iHEAL implementation and scale up.

## Introduction

More than four out of ten Canadian women report having experienced intimate partner violence (IPV) at some point in their lives [1]. In addition, people who occupy more marginalized positions in society (e.g., those who are younger, live with a disability, those who identify as Indigenous, those who identify as a member of a gender or sexual minority group) are more likely to experience violations of power, including IPV, and have fewer options for countering the violence. These disadvantages are examples of structural violence, or the assertion that inequities are inherent in the way society is organized, causing structurally-rooted harms [2]. A substantial body of evidence documents the significant toll of IPV on women’s sense of safety, security, relationships, finances, and mental and physical health [3,4]. For many women, these health, social, and economic impacts persist over time, including after separation from the abusive partner [5–7].

Women with histories of IPV access services (including health care) at higher rates than the general population [8]. Yet most services tend to focus on addressing immediate *crises* and often consider IPV to be an *episodic* rather than *chronic* issue, resulting in a ‘mismatch’ between the services offered and the needs of women who are seeking support to mitigate the ongoing effects of IPV on their lives. Although there has been significant growth in the development and testing of IPV interventions in the past decade [9], a gap remains in the identification of effective interventions that focus on assisting women to navigate the multiple, often intersecting issues, inclusive of ongoing health and safety challenges related to experiencing IPV.

**Fig 1:**
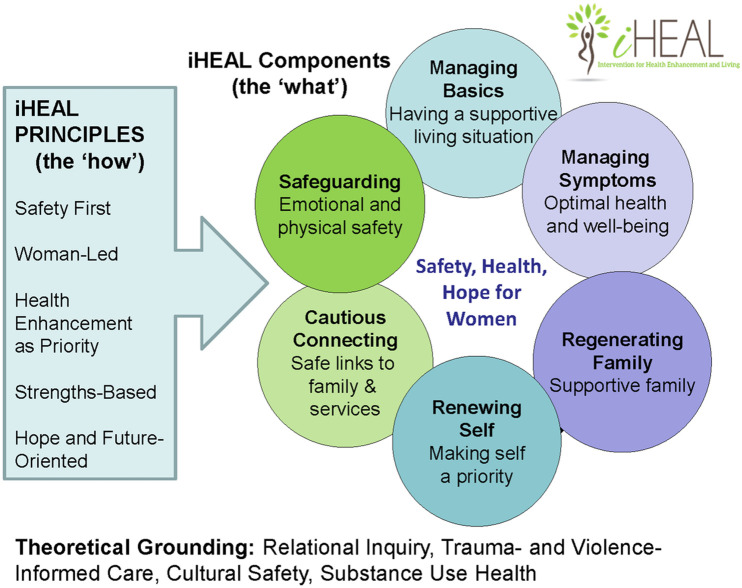
Principles, components and theoretical grounding of iHEAL. Source: Adapted from Ford-Gilboe M, Varcoe C, Scott-Storey K, Browne AJ, Jack SM, Jackson K, et al. Longitudinal effectiveness of a woman-led, nurse delivered health promotion intervention for women who have experienced intimate partner violence: iHEAL randomized controlled trial. BMC Public Health. 2024 Feb 7;24(1):398.

The Intervention for Health Enhancement and Living (iHEAL) is an evidence-based, comprehensive, woman-led health promotion intervention for women with experiences of IPV [10–13]. The intervention is based on principles of trauma-and violence-informed care, relational inquiry, cultural safety, harm reduction, and qualitative grounded theory research that explores the health promotion priorities of women who had separated from an abusive partner and were trying to create a new life in the context of *intrusion* (i.e., external interference in their lives) [7,10,14]. This evidence base is operationalized as a set of five principles that guide all interactions with women, and six components that reflect the boundaries of the intervention (see Fig 1). Within this framework, specially educated and trained iHEAL Registered Nurses draw on their knowledge and expertise to assist women to address diverse issues that affect their health, safety, and well-being in 10–18 visits over approximately six to eight months. Designed to be inclusive, a key feature of iHEAL is the flexibility to tailor the program to a woman’s individual needs and priorities, and to the community in which she lives. The intervention is delivered in collaboration with local services, according to the woman’s wishes, as the aim is to complement and extend, rather than duplicate, existing services.

iHEAL nurses are Registered Nurses who hold a minimum of a Baccalaureate degree. To prepare them to offer iHEAL, they complete 60 hours of initial education (offered by a team of iHEAL Educators via online modules and live training sessions) and then work in the context of a team to offer the intervention to women who voluntarily enroll on their own or via referral. Nurses are supported by a supervisor who facilities team meetings, supports ongoing professional development, encourages debriefing as needed, provides clinical supervision, and helps the team connect to new resources or learning to strengthen supports offered to women. Written program requirements and other resources (e.g., a 200-page Practice Guide for Nurses, a Women’s Guide) provide additional details about iHEAL and practical guidance for implementation. An iHEAL Consultant supports the tailoring of iHEAL to the local and organizational context, ongoing education, and practice development for program delivery. Additional details of the iHEAL education and delivery model can be found elsewhere [11].

In a series of feasibility studies using pre-post single group designs, iHEAL demonstrated high acceptability and safety among women from diverse backgrounds (inclusive of Indigenous women and Francophone women) [12,13,15]. Further, in a randomized controlled trial of 331 Canadian women in the transition of separating from an abusive partner [11], when compared to usual care, iHEAL was found to be more effective in improving women’s mental health, quality of life, and confidence in managing daily life while concurrently reducing the severity of violence they experienced. These positive effects were seen immediately post-intervention and sustained for one year after the iHEAL intervention ended. Notably, iHEAL is one of only a few interventions for women experiencing IPV offered by health professionals that is supported by evidence of benefits for both women’s safety and their health over a sustained period of time.

The iHEAL trial was conducted under ideal conditions, with nurses and supervisors recruited, trained, and supported by the Research Team as they offered the program, and not part of usual health care services. Whether it is feasible to translate complex interventions, like iHEAL, with fidelity into ‘messy’ real-world service settings and maintain their benefits is a critical challenge [16,17]. As complex adaptive systems, health care organizations are dynamic, relational, interconnected networks that respond to shifting priorities and external pressures in ways that are constantly evolving, and sometimes unpredictable [18]. When implementing complex interventions that challenge models of ‘usual care’, as iHEAL often does, the organizational context has a powerful influence on whether and how these interventions are taken up with fidelity [19]. Considerations such as leadership, the fit between the intervention and organizational policies and processes, and, in the case of iHEAL, organizational norms and expectations about the scope and breadth of nursing practice and understanding of IPV within organizations could affect whether and how an intervention can be offered with fidelity in a specific context.

## Aim and research questions

The aim of this study is to understand whether and how iHEAL, a complex intervention shown to be effective when tested under ideal conditions, can be offered by nurses working in diverse community-based health care organizations in ways that are locally appropriate and retain iHEAL’s impacts for women, organizations, and communities. We will focus specifically on the *processes and resources* needed to implement iHEAL with fidelity and to support *sustainability.* By understanding complex implementation processes in context, we expect that the findings will provide critical insights about the diverse factors that shape implementation across varied health care settings, including tensions related to fidelity, and suggest additional resources or supports needed to strengthen future implementation of iHEAL.

**Five Research Questions will be addressed:**

1. How can iHEAL be implemented with fidelity in varied community-based health organizations located in 3 provinces? What challenges fidelity and how can these issues be addressed and mitigated? [*Processes of Implementation and Fidelity]*
2. To what extent are organizations able to reach and engage women, including those who face access barriers and those who have had negative prior experiences in health care? What facilitates or hinders engagement? [*Reach and Engagement*]
3. Are benefits to women, including improvements in health, quality of life and other outcomes, maintained when iHEAL is implemented in real-world settings? Under which conditions? [*Effects/Benefits*]
4. What are the benefits or ‘value-added’ to organizations that implement iHEAL and/or to agencies or groups within local communities? [*Benefits to Organizations/Communities*]
5. What system and organizational resources are needed to support the effective and sustainable implementation of iHEAL? [*Sustainability*]

## Theoretical approach and study design

Our approach to this implementation study is informed by the Active Implementation Frameworks (AIFs), an established Implementation Science approach that is particularly useful in guiding the implementation and evaluation of complex, tailored health and social services and programs, such as iHEAL [20,21]. AIFs emphasize the iterative and evolutionary process of implementation and evaluation research activities; the need to attend to both process and outcomes, with attention to implementation drivers and tailoring activities to local contexts; and the importance of engaging key stakeholders throughout the process, including when identifying lessons and implications for sustainability.

These features have been integrated into our implementation study design, across three phases: 1) Initial planning and program delivery on a small scale, prior to this study; 2) formal planning, implementation and evaluation over ~2.5 years; and 3) consolidation of findings and development of guidance for future implementation of iHEAL. For example, we will summarize and share descriptive research data with organizations at planned intervals (e.g., mid-study) to support local refinements (or *improvement cycles*). We will also draw on concepts from AIFs (e.g., implementation drivers, fidelity) as a theoretical lens for analyzing and making sense of the data collected. Development of guidance materials for future implementation will be completed in collaboration with implementation teams in each organization.

## The context of implementation

Three community-based health services will collaborate on this study, each of which is in the early phases of planning and/or offering the iHEAL program as part of its services. Table 1 provides details about each of the organizational contexts. Of note, and consistent with the Canada Health Act [22], each organization offers a range of accessible, publicly-funded health services and programs. These organizations have also made an explicit commitment to equity and serving people marginalized by structural inequities; all serve high proportions of women facing structural barriers to healthcare and increased risk of violence. Given the sites are located in three different provinces, the provincial funding that each organization receives will vary, as health care delivery in Canada is largely a provincial responsibility, supported by federal transfers. There are additional differences across these organizations related to organizational history, size and populations served, geographic location (small, medium and large urban contexts), as well as staff mix and funding sources. These variations across implementation sites will provide an important opportunity to consider how different organizational and contextual features shape iHEAL implementation, with potential implications for sustainability and future scale-up.

**Table 1:** Organizational characteristics and implementation models.

	Site A	Site B	Site C
**Type of Organization**	Indigenous health and wellness cooperative *Programs*: member-driven including cultural wellness, primary care, harm reduction, food and nutrition, peer-led wellness, case management and outreach	Public Health Unit *Programs:* health promotion and disease prevention (universal and targeted) in priority areas such as sexual and reproductive health, child/youth health, healthy start, injury prevention	Nurse-led Primary Health Care center *Programs:* clinic-based ambulatory care focused on illness and injury prevention, women’s health, chronic disease management, sexual health, laundry, hygiene and outreach supports
**Geographic Context**	Urban inner city: population > 2 million (British Columbia)	Urban-rural region: population ~500,000 with 10% rural (Ontario)	Small urban: population ~101,000 (New Brunswick)
**Populations Served**	Members experience complex health and social challenges linked to interpersonal and structural violence, including racism and other forms of discrimination	Proportionate universalism approach: programs for all and for priority populations based on need (including those at risk of poor health or developmental outcomes)	Clients who face barriers to health services, including immigrants and refugees; people experiencing problematic substance use, mental health issues and/or housing insecurity
**History and Governance**	Newer non-profit organization and registered charity governed by a volunteer Board (minimum 50% Indigenous Elders) and an Executive Director reporting to the Board.	Mandated agency with an appointed Board of Health that oversees budget, policy, and implementation of services/programs. A CEO, Medical Officer of Health and Chief Nursing Officer report to the Board.	A community health centre governed by a provincial Health Authority (Board of Directors) under a joint MOU with the local university. The VP Community reports to the CEO (reporting to the Board).
**Funding Sources**	No core program funding. Short-term contracts from local, provincial, federal governments and reimbursement of fees for physician services.	Core provincial and municipal (local) health funding for mandatory programs, augmented by short-term contracts for other priorities.	Core provincial health funding, augmented by short-term contracts for other priorities.
**Staff Mix and Compensation**	Interdisciplinary staff mix including physicians, nurses, social workers, Elders, peer supports, and others Mix of salaries and hourly wages; staff are not unionized	Interdisciplinary mix including Registered Nurses, dental hygienists, public health inspectors, nutritionists, and epidemiologists Staff are salaried; nurses are unionized	Interdisciplinary mix including Physicians, Nurse Practitioners, Registered Nurses, Licensed Practical Nurses, social workers, respiratory therapists, and dieticians Staff are salaried; nurses are unionized
**iHEAL Delivery Model**	iHEAL + Group: 2 RNs (.8 FTE each) delivering iHEAL to a caseload of 10–12 women each Group-based cultural activities led by Indigenous Elders and supported by a trauma counsellor offered alongside nurse visits (called Reclaiming our Spirits, ROS)	iHEAL Only: 1 RN (full-time) delivering iHEAL to a caseload of 16–20 women Concurrent Delivery: Up to 20 RNs offering iHEAL to women on their caseloads in 2 home visiting programs for young families (pregnancy to preschool years), iHEAL caseload 1–2 women/year	Concurrent Delivery: 5 RNs (full-time) each with 20% time allocated to delivery of iHEAL, with other responsibilities providing ambulatory care services; iHEAL caseload of ~5 women per nurse/year

### Initial planning and program delivery (Phase 1)

**Fig 2:**
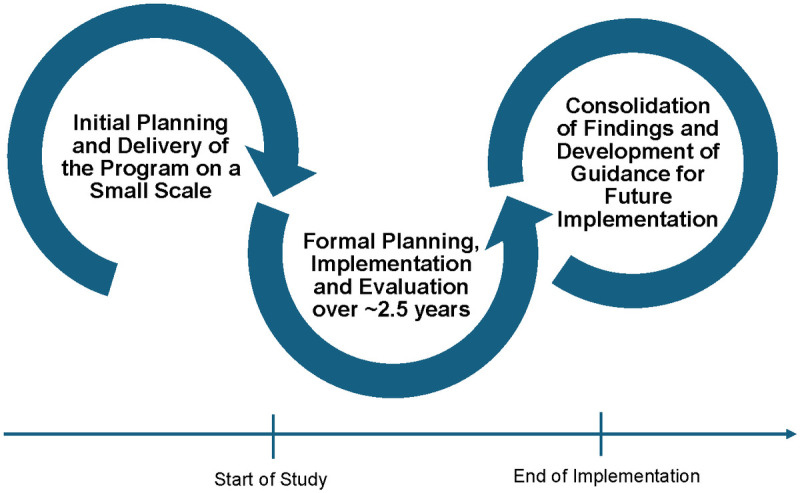
Phases of implementation.

Fig 2 outlines the phases of this research. Prior to initiating this study, each organization began planning for the delivery of iHEAL, with a small group of nurses and managers participating in iHEAL education who subsequently started to offer the program on a small scale in collaboration with the Research team. At this time, each organization identified the model they would use to offer the iHEAL program if funding could be obtained and estimated the number of women who could be served based on these models (see Table 1). Thus, the approaches to offering iHEAL will vary across organizations, with models where nurses offer iHEAL only and those where nurses will offer iHEAL as part of a larger workload, concurrent with responsibilities for other programs or services (i.e., home visiting programs, ambulatory care). In one site primarily serving Indigenous people, the plan is to offer iHEAL to women and also invite them to take part in a weekly group led by Elders and Knowledge Keepers designed to share cultural teachings and promote women’s safety, connections and peer support. This approach was previously developed and tested by members of the Research Team [12,15] and named ‘Reclaiming our Spirits’ by the women who participated.

### Formal implementation and evaluation plan (Phase 2)

**Fig 3:**
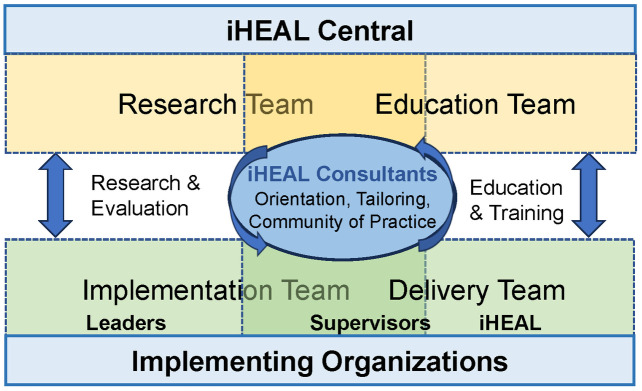
Structure supporting implementation, education and evaluation.

Formal implementation in each organization will be supported by a series of interacting teams with specific functions (see Fig 3). Within *iHEAL Central*, academic team members serve on the *Research Team*, *Education Team,* and/or as *iHEAL Consultants*. Within health care organizations, a *Local Implementation Team* will be responsible for planning and oversight of the program, while a *Program Delivery Team* (a supervisor and iHEAL nurses) will be responsible for offering iHEAL to women. The iHEAL Consultants are part of iHEAL Central but sit at the interface of various teams, and are a consistent mechanism that supports communication, connection, preparation, tailoring and delivery of the iHEAL, working across the Implementation Team and Program Delivery Team.

#### Local implementation teams.

In the initial planning, a Local Implementation Team will be set up to guide planning and delivery of the iHEAL program at each organization. Each team will include members of the organization, such as (at minimum) a manager/supervisor responsible for oversight of the program and supervision of nurses*,* Registered Nurses who will offer the program*,* and an iHEAL Consultant who will provide guidance and support to assist organizations with planning and roll out of the program (up to 10 hours per month).

These Local Implementation Teams will make decisions about how to *operationalize* the iHEAL program requirements to align with local priorities and contexts and to promote fidelity. Activities include, for example, developing and enacting plans for recruiting and enrolling program participants; support and mentoring of iHEAL nurses; establishing or strengthening collaborations with community agencies to support implementation; identifying methods of gathering and tracking program statistics, including modifying or adapting Electronic Medical Records (EMRs); and setting up processes to engage in quality improvement to refine the program over time. Local Implementation Teams will meet regularly to review their progress, identify challenges and successes, and adjust their initial plans to address issues and strengthen program delivery.

#### iHEAL education.

All staff will be offered an orientation to iHEAL by the Research Team. In addition, nurses will be required to complete ~60 hours of standardized education offered by the iHEAL Education Team using a combination of individual online and in-person group delivery, with interactive activities, assignments, and group discussions. The online education will focus on increasing foundational understanding of iHEAL’s five principles and six components, its conceptual foundations (i.e., cultural safety, trauma-and violence-informed care, relational inquiry, substance use health) and common issues faced by women who have experienced IPV (e.g., safety and IPV dynamics, health effects of IPV, help seeking and barriers to support). Nurses will also complete standardized online training in the use of the Danger Assessment [23], a lethality assessment incorporated into iHEAL, and San’yas Indigenous Cultural Safety training [24] as a foundation for appreciating the impacts of colonization on Indigenous women in Canada and considering how to offer iHEAL in ways that are safe and appropriate. During in-person sessions, nurses will be supported to learn about and connect with Indigenous people they serve. Building on this understanding, in-person sessions will focus on preparing nurses to deliver iHEAL with fidelity. These sessions will focus on the application of iHEAL principles and components and reinforce the importance of taking a woman-led, non-judgmental approach. Given the critical role of managers and/or supervisors in supporting the iHEAL program, they will also be invited to take part in this education, along with an additional session focused on supervision of nurses and program start up.

#### Program delivery.

After completing the required education, working as a Program Delivery Team, nurses and supervisors will offer iHEAL over ~2.5 years. Organizations will activate their plans for promoting the program and inviting women to take part, enrolling women, and assigning a nurse to work with each woman. Initially, each nurse will work with 1–2 women and gradually build their caseloads as their confidence and competence improves. Nurses will work within the scope of their professional standards of practice, as established by the province in which they work. All staff will be expected to follow the policies of their organization, including, for example, those that address the negotiation of safe, private spaces for visits, and documentation practices. However, iHEAL Consultants will work with organizations to review and revise or develop policies to optimally support implementation (e.g., policies regarding missed appointments, policies regarding child protection protocols).

During the period of program delivery, Program Delivery Teams will meet every 1–2 weeks for the purposes of case review, problem-solving, sharing resources, and debriefing. These meetings will be arranged by the supervisor and held in person or virtually, according to the preference and practice of the organization. The iHEAL Consultant and/or invited experts (e.g., those with knowledge of chronic pain management, or legal systems women encounter) may also be invited to take part in these meetings, depending on the learning needs participants identify. iHEAL Program Delivery Teams will engage in a naturalistic process of refining program delivery over time based on their experiences of working with women, while concurrently considering fidelity, organizational structure, and local needs.

The iHEAL Program Delivery Teams from each organization will also be invited to participate in a virtual *Community of Practice* (COP) ~every 4–6 weeks. These sessions will be hosted by the iHEAL Consultants and will bring together teams from all sites for discussion, sharing of learning and innovations, and ongoing education and professional development. Consistent with principles of adult learning and standards for communities of practice, the process used in these meetings will prioritize engagement and interaction, with topics for discussion identified by the participants.

### Consolidation and guidance development (Phase 3)

Toward the end of the implementation period, we will shift focus to identifying and mobilizing consolidated lessons and implications from this research and generating practical guidance for future implementation and sustainability of iHEAL. This work will be done in collaboration with implementing organizations and other external stakeholders.

Planned activities are part of the project’s Integrated Knowledge Mobilization approach. With our site partners and stakeholders, the Research Team will review and discuss research findings within and across sites as a means of identifying key lessons and implications for the development of resources and guidance to support future implementation and sustainability of iHEAL (locally and for wider scale up beyond initial sites). We will also seek initial input on draft implementation resources and guidelines and use that feedback to evolve these resources.

Our aim is to develop and consolidate a set of implementation resources and guidelines that will comprise an implementation package (a main deliverable of this study). Informed by the Implementation Science literature and findings from this study, the guidance resources will cover all phases, from planning to full implementation. We anticipate that the final package will include: a) *‘Pre-Commitment’ Package* for organizations interested in iHEAL, including an asynchronous orientation webinar; b) *Site Assessment/Negotiation Materials*, such as: MOU template, fidelity requirements, partnership agreements, organizational capacity assessment, nurse-supervision arrangements, educational requirements (at the level of nurses and the organization), budget template; and c) *Comprehensive Guidelines* for delivering iHEAL in different contexts, including a CQI framework (i.e., indicators, tools, and process), sample promotional materials, and access to ongoing iHEAL supports and updates. We will also update and/or evolve iHEAL education materials and resources based on feedback solicited from Local Implementation Team members and Program Delivery Teams. All documents will include considerations for sustainability, and iHEAL Consultants will work with each site to develop their own sustainability plan, potentially building on an early blueprint/framework for implementing ROS in diverse contexts so that our sustainability plan includes guidance toward working in Indigenous-specific contexts.

Specific end of study Knowledge Mobilization (KMb) activities will include: hosting knowledge sharing forum(s) in each province for those who participated in the study and a national forum with policy actors focused on sustainability and scale- up; creating brief summaries of results (e.g., policy briefs, videos, infographics, other communication strategies) tailored for different audiences; and identifying “iHEAL Champions” for future sustainability.

## Methods

We will use a convergent, parallel, mixed methods design to gain a deep and comprehensive understanding of the processes and context of implementing iHEAL, with particular emphasis on the organizational structures and resources required for fidelity and sustainability, as well as impacts for both women who access this program and for organizations that offer iHEAL.

Starting with the period of formal implementation and evaluation, we will collect varied sources of qualitative and quantitative data as we work in partnership to support and study implementation in three diverse community-based health care contexts, periodically sharing descriptive summaries of the data collected with organizations to assist them in refining iHEAL delivery. Aligned with the research questions outlined above, in-depth qualitative data will be used primarily to explore the implementation process, fidelity of delivering iHEAL, structures required to support the program, and other factors related to the organizational and implementation context, while quantitative data will be collected to understand trends in program engagement and impacts/benefits of iHEAL for women.

Since each organization began initial planning and service delivery on a small scale prior to the start of this study, data collection will begin by *retrospectively* exploring each organization’s experiences of planning for and initially delivering iHEAL, including capturing any adaptations made to fit with the local context, as well as *current* delivery issues. Subsequently, data collection will shift to *prospectively* describe implementation processes as they roll out in each organization, along with associated outcomes, including women’s experiences of the intervention, over a 2.5-year period. Recruitment and data collection are in progress, with varied start and end dates based on participant group and type of data collected (see ‘Progress to Date’ for details).

### Sampling and recruitment

To generate a comprehensive and holistic understanding of the processes of implementation and delivery, individuals from 4 groups at each implementation site will be invited to take part in this study: a) organizational leaders, b) implementation/delivery team members, c) women who have engaged in iHEAL, and d) representatives from community organizations or groups that have supported or collaborated with the program. Program administrative data (e.g., documentation of fidelity, number of women in the program, referral sources) and meeting notes will be used as additional sources of data.

#### Organizational leaders, implementation/delivery team members, community organizations.

In each organization, a designated project contact will share the names and email addresses of 3–5 organizational leaders (i.e., program managers, administrators, board members, and/or clinical leaders) who have an interest in, or some responsibility for, iHEAL, along with all iHEAL Implementation/Delivery Team members. The composition of the Local Implementation Team and Delivery Team at each site will depend on the organization’s unique structure and implementation model. Therefore, individuals in a number of roles will be invited to participate, including: iHEAL nurses, supervisors, managers, consultants, and others involved in delivering the program. New leaders or staff who join Local Implementation or Delivery Teams part way through the study will be invited to take part in the next set of interviews (i.e., we will sample team members who are willing and able to participate at each point, rather than following individuals over time). Additionally, a project contact from each organization will also provide contact information for 3–5 representatives from community organizations identified as being involved in some way with the iHEAL program (e.g., by making or accepting referrals) and/or those who have an interest in the program from a systems perspective (e.g., decision-makers in a health authority).

A member of the Research Team will send each potential participant an invitation to participate, with 2 follow-up messages for those who do not respond. Written informed consent will be obtained electronically; those who wish to participate will be sent a secure link with the Letter of Information and Consent to review and sign if they agree to participate. If electronic consent cannot be completed, verbal consent will be obtained prior to an interview taking place. The estimated sample sizes for each participant group across all sites are: 9–15 organizational leaders; up to 30 Implementation/Delivery Team members; and 9–15 representatives of community organizations or groups.

#### Participants in the iHEAL program.

Adult (18 years or over), English speaking women who enroll in iHEAL at one of the study sites will be invited to participate in pre-post intervention surveys, and/or qualitative interviews, with 3 follow-up messages sent for all research activities to women who do not respond to the initial invitation.

***Pre-Post Intervention Surveys:*** All women who enroll in the iHEAL program will be provided with information about participation in the surveys when they begin the program by a staff member in the organization who will use a standard script. When women are interested in participating or learning more about this research, with the woman’s permission, the staff member will collect and provide her safe contact information (i.e., first name, phone number, and/or email address) privately and securely to the Research Team. A Research Coordinator will contact each participant to provide information about the study, answer questions, obtain informed consent and arrange for the first survey. A Letter of Information and Consent will appear at the beginning of the pre-intervention survey. To proceed, the woman must give her consent by checking a box.

A total of 400 women are expected to participate in iHEAL across the sites, all of whom will be invited to take part in surveys. We estimate that 70% of women will agree and complete the first survey (i.e., 280 women), with 20% attrition for the second survey, bringing the potential sample to 224 women. The sample size needed to detect pre-post program differences (using paired t-tests) in the two primary outcomes from the iHEAL trial is 220 for PTSD symptoms and 63 for Quality of Life, using trial effect sizes (.36 for QOL, −.19 for PTSD symptoms) with a power of.80 and alpha of.05, calculated using G Power [25].

***Qualitative Interviews*:** A purposive sample of up to 60 women (~20 per site) will be invited to take part in a qualitative interview as they complete the iHEAL program. Women will be able to indicate their interest in an interview in two ways: 1) by responding to a question on the post-intervention survey, or 2) by indicating their interest to their nurse who will provide information about the interview during the woman’s last iHEAL session. This strategy will allow women who do not complete one or both surveys to participate in an interview. In this case, the nurse will provide the woman’s contact information to the Research Team using the same process outlined for surveys. Women who indicate their interest in an interview will be contacted by a Research Coordinator who will provide information, answer questions and invite participation. Maximum variation sampling will be used to identify a sample that reflects the diversity of participants’ socio-demographic characteristics (for those who have completed a survey and provided this information). Women will receive the Letter of Information and provide written consent electronically through a secure link sent to them by the Research Coordinator, or if this is not possible, verbal consent will be obtained prior to the interview taking place.

### Data collection

The data collection plan includes a mix of quantitative (administrative data, surveys) and qualitative (interviews, meeting notes) data from organizational leaders, implementation team members, community or external partners/stakeholders, and women participating in iHEAL. The anticipated timing of all data collection activities is outlined in Table 2.

**Table 2:** Data collection plan.

Qualitative Data	
Data Type and Source	Proposed Timing
Interviews: Implementation/Delivery Team Members x3	Baseline: start of formal implementation (retrospective, current) Mid-study: ~ 1.5 years after baseline End-of-study: ~ 2.5 years after baseline
Interviews: Organizational Leaders x2	Baseline: start of formal implementation (retrospective, current) End-of-study; ~ 2.5 years after baseline
Interviews: Women Participating in iHEAL x1	Post-intervention: ~ 6–8 months after women start the program
Interviews: External Community Agencies x1	End-of-study: ~ 2.5 years into formal implementation
Meeting Notes	Continuously collected
**Quantitative Data**	
**Data Type and Source**	**Proposed Timing**
Surveys: Women Participating in iHEAL x2	Pre-intervention: when women start the program Post-intervention: ~ 6–8 months after start
Program Administrative Data	Continuously collected

#### Interviews.

In keeping with the principle of emergent design, all interviews will be semi-structured and follow a flexible interview guide that will be refined as the project progresses. Interviews will last between 30 and 60 minutes, and will take place in person, by phone, or videoconferencing, depending on the participant’s preference. Interviews will be audio recorded, with the participant’s consent, and transcribed for analysis. Participants will be able to skip any questions they do not wish to answer and/or end the interview at any time. Qualitative interviews will be conducted with 4 groups of participants:

1. *Organizational Leaders:* Organizational leaders from each partner site will be invited to participate in two interviews: Baseline and End-of-study. Baseline interviews will explore organizational leaders’ roles and experience in implementing the iHEAL program, the issues they or their teams have experienced and how these have been addressed, and issues they anticipate moving forward. Additional questions will focus on factors that led to the organization deciding to offer the program, program fit with the organization, and organizational level issues and strategies to support iHEAL implementation.

End-of-study interviews will focus primarily on the challenges of offering iHEAL over time, key lessons, and organizational practices and resources that may be necessary for continued/future implementation and scale-up of the iHEAL program. Additional questions will focus on the benefits of the iHEAL program to the organization and to their partners and community, and potential opportunities for sustainability and scale up.

2. *Implementation/Delivery Team Members:* All members of each organization’s Implementation and Delivery Teams will be invited to participate in three interviews: Baseline (immediately following study launch), Mid-study (about half-way through the implementation phase), and End-of-study (at the end of the implementation phase).

Similar to baseline interviews with organizational leaders, baseline interviews with Implementation/Delivery Team members will explore each participant’s role and initial experiences in planning and offering the iHEAL program, the issues they or their teams have experienced and how these have been addressed, and issues they anticipate moving forward.

Mid-study interviews will explore issues or challenges that Implementation/Delivery Team members have encountered in delivering the iHEAL program as it has become more established, strategies used to address challenges, changes to iHEAL program model or service delivery, decision-making processes, and resources needed to offer the program successfully.

End-of-study interviews will focus on similar issues covered in organizational leader interviews, with a primary focus on understanding the challenges of offering iHEAL over time, key lessons, and organizational practices and resources that may be necessary for continued/future implementation and scale-up of the iHEAL program.

3. *Representatives of Community Organizations:* Concurrent with end-of-study interviews outlined above, brief interviews with community organizations will focus on their impressions of the program, its fit with the local services landscape, and sustainability considerations.
4. *Women Enrolled in iHEAL:* Interviews with women who take part in the iHEAL program will focus on their general experiences of iHEAL, including: facilitators and barriers to participation; impacts of iHEAL on their lives (if any); how their particular situation (e.g., geographic location, whether women are mothering or not, whether living with an abusive partner or not) affected their experiences of iHEAL; and any recommendations they have for strengthening the iHEAL program.

At the end of the interview, the interviewer will review signs of a stress response and talk with the participant about how she can manage these if they arise. If it is safe to do so, the interviewer will send the participant a document outlining these stress responses and management strategies, as well as a list of local resources/services she can access should she need further support.

#### Pre-post intervention surveys with women.

Surveys will be administered online using Qualtrics® at two points in time: 1) at program enrollment (pre-intervention), and 2) ~6–8 months after initial enrollment (post-intervention) when women will have completed the program or will be in the final stages of completion. To reduce barriers to participation, women will have the choice of completing the survey online on their own (*self-completion*) or in person, with assistance from a Research Coordinator (*assisted completion*). Women who complete the survey on their own will receive a link via email, using a safe email address. If a woman does not have a safe email address, the Research Coordinator will help her set up a free account. Women will have three weeks to complete the survey and will be sent up to 3 email reminders to complete the survey.

For those who wish to complete the survey with assistance, the Research Coordinator will schedule a time to meet with the woman in a safe, private location (such as a library or service agency). In-person surveys will be facilitated by a trained researcher who will enter responses into the Qualtrics survey using a tablet device. This tablet interface will allow the participant and researcher to view questions and select responses together on the screen.

The surveys were designed with readability, accessibility and safety in mind, based on questions used in the iHEAL trial. The final page of each survey (pre- and post-intervention) includes information about the signs of a stress reaction and ways of managing any distress women may feel after the survey, including how to access local services. We have used this form in many previous studies as a way of helping women anticipate the possibility of distress and how to manage it safely.

Both pre- and post-intervention surveys will include key outcome measures used in the previous iHEAL trial, assessed at baseline (pre-intervention) and again at program completion (post-intervention). These self-report measures include:

1. *CESD-R Depression Scale:* 20-item self-report measure of depressive symptoms in community samples, including the probability of meeting criteria for major depressive disorder [26]. Women rate how often they have experienced each symptom in the past week using 5 options ranging from ‘not at all or less than 1 day’ to ‘nearly every day for two weeks’. Total and subscale scores are computed by summing responses to applicable items, with higher scores indicative of greater symptomology.
2. *PCL-C-SF for PTSD*: 6-item self-report measure of symptoms of posttraumatic stress disorder (PTSD) derived from the full 17-item checklist (PCL-C). The short-form version of this scale has demonstrated adequate validity and reliability in primary care settings [27]. Women rate how often they experience the listed responses to stressful life experiences on a 5-point Likert scale ranging from ‘not at all’ (1) to ‘extremely’ (5). Scores range from 6 to 30, with higher scores indicating greater symptomology. A score greater than 14 is considered to a positive indicator for PTSD.
3. *Quality of Life Scale:* 9-item self-report measure developed to assess quality of life across domains important to women who have experienced IPV [28]. Items are rated on a 7-point Likert scale from ‘extremely pleased’ (1) to ‘terrible’ (7), with total scores computed by reverse-scoring and summing responses to all items; higher scores are indicative of better quality of life.
4. *Confidence in Managing Daily Life Scale:* 7-item self-report scale, adapted from the longer 10-item version used in the iHEAL trial. Developed using Bandura’s methodology, items measure women’s confidence (self-efficacy) in engaging in a set of actions that fit with key goals of iHEAL on 100 mm visual analogue scales, with anchors of ‘not at all confident’ and ‘completely confident’. Scores range from 0 to 100, with higher scores representing greater confidence in managing daily life.
5. *CAS*_*R*_*-SF:* 16-item summated rating scale designed to capture the severity of physical, sexual, and psychological abuse, including coercive control, from a current or former intimate partner [29]. For each item, women are asked to indicate whether they have ever experienced the abusive act, and if so, to rate the frequency of experiencing it in the previous 6 months on a 6-point Likert scale with options of ‘never’ (0) to ‘daily or almost daily’ (5). Total summed scores range from 0–80, with higher scores indicative of greater severity of IPV.
6. *Chronic Pain*: 7-item self-report measure of chronic pain intensity and disability, including 4 “grades” that capture level of disability (from ‘no’ to ‘high’) [30]. The disability score reflects the impacts of chronic pain on an individual’s life and functioning. It is computed as the mean score of 3 items that ask women to rate the degree to which pain interferes with aspects of their life on a 10-point scale from ‘does not interfere’ (0) to ‘completely interferes’ (10).

In addition to these scales, the pre-intervention survey will include basic demographic questions (to enable sample description). The post-intervention survey will include questions about women’s level of engagement in iHEAL, their experience engaging in the program, barriers to engagement or participation, and questions focused on safety in iHEAL visits and satisfaction with the program.

#### Program administrative data and meeting notes.

1. *Program Data:* Throughout project implementation, each implementing organization will collect administrative program data. These data will focus on program delivery, including number of women in the program, number and type or method of visits, length of time in the program, fidelity (e.g., whether each intervention component was addressed), and summarized de-identified demographic data for women taking part in iHEAL (e.g., ethnocultural identity, Indigenous identity, living in a rural area). These data will be shared with the Research Team every 6 months for women who have completed enrollment during that time period. Information that could identify participants will not be shared.
2. *Meeting Notes:* High level notes capturing implementation challenges and strategies discussed at meetings with leaders, Implementation Teams, or Delivery Teams will be taken by a member of the Research Team using a standard template. These notes will not identify individuals, nor will these sessions be recorded and transcribed. In the analysis, these notes will provide supplementary information about broad implementation issues and challenges and how these were negotiated within and across sites. Information about the collection of meeting notes will be provided in the information sessions hosted at the project launch.

#### Data management plan.

A variety of strategies will be used to promote privacy and confidentiality of collected data. This includes: each site assigning a unique study ID for each participant; maintaining a master list with these IDs and personal identifiers (e.g., names, emails, phone numbers) accessible only to the Research Coordinator and Primary Investigator at each site and stored securely behind firewalls and separate from any additional personal identifiers collected; and de-identifying all data prior to analysis. Personal identifiers will be used to conduct virtual or phone interviews and for recruitment of participants into the study. Additionally, the Research Team will use the woman’s email address (for online surveys) or study ID (for assisted surveys) to match each woman’s pre-intervention and post-intervention survey in order to examine changes in key outcomes over time. All de-identified data will be stored on a secure study-designated Microsoft Teams Channel hosted at the Western University behind institutional firewalls, or in a locked cabinet at Western University. In alignment with Western University’s ethics requirements, data will be securely stored for at least 7 years and participants will be informed of these processes and the potential for future use of the data.

#### Progress to date.

Recruitment and data collection timelines vary by participant group and type of data collected (see Table 3). All data collection is expected to be complete by October 1^st^, 2025. We anticipate that first results will be ready for publication in January or February 2026.

**Table 3:** Study recruitment and data collection timelines.

Participant Group [Data Type]	Recruitment		Data Collection	
	Start	End	Start	End
Organizational Leaders [Interviews x 2]	December 2022	December 2023	December 2022	May 2025
Implementation/Delivery Team Members [Interviews x 3]	December 2022	April 2025	February 2023	May 2025
External Stakeholders [Interview x 1]	June 2025	October 2025	June 2025	October 2025
Women participating in iHEAL [Pre-post Surveys]	April 2023	October 2025	April 2023	October 2025
Women Participating in iHEAL [Post Interviews]	August 2023	October 2025	August 2023	October 2025
Program Administrative Data	N/A	N/A	November 2022	October 2025
Meeting Notes	N/A	N/A	November 2022	October 2025

### Data analysis plan

Consistent with the iterative design of this project, we will conduct initial descriptive analysis as data are collected. This will enable the Research Team to share two brief summaries of key issues with implementing organizations to help them fine-tune initial delivery of iHEAL: a) *Consensus Summaries* will outline the history and context of offering iHEAL in the organization, based on retrospective accounts collected during baseline interviews; and b) an *Interim Report*, will describe some key implementation issues identified during full implementation, based on initial descriptive analysis of interview data, meeting notes, and/or administrative data. All analyses will be conducted first by site and then across sites.

#### Qualitative data.

We will organize and analyze transcribed, de-identified, and cleaned interview data and meeting notes using the desktop version of NVivo, a qualitative data management software program. In the early phases of analysis, we will employ Rapid Qualitative Team-based Analysis (RQA) [31–33] to describe key aspects of the implementation (e.g., delivery model, mentoring and support, funding and sustainability, staffing and recruitment, etc.), along with variation in fidelity according to iHEAL principles and requirements, and the fit of iHEAL within the organizational context. From this analysis, we will summarize key issues that are identified within and across organizations.

As the analysis proceeds, we will take a more interpretive approach that draws on Reflective Thematic Analysis [34,35] to further explore and describe key issues or areas of interest identified in the initial RQA, guided by our research questions. We expect that key issues will pertain to implementation processes, impacts, strategies for local tailoring, facilitators of and challenges to fidelity, considerations for scale-up and sustainability, and impacts for women. However, the flexible approach and design of our research will allow for the exploration of additional areas or themes identified as the analysis proceeds.

#### Quantitative data.

Program administrative data and survey data will be summarized using descriptive statistics, overall and by site. The administrative data will be used to create an implementation profile for each site, and to contextualize the qualitative data collected in interviews and through meeting notes. Reach will be measured by the number of women invited to participate in iHEAL and engagement will focus on patterns of women’s visits, including the number or frequency of visits, program completion, length of time to complete the program, and visit type (e.g., in person, by phone, etc.). Fidelity will be measured by whether nurses are addressing each iHEAL component with women, and completing any required activities, with supplementary qualitative data from interviews and women’s surveys also used to assess fidelity to the iHEAL principles and theoretical foundations.

Short-term changes in outcomes for women participating in iHEAL will be examined using paired t-tests to determine if there are significant improvements in outcomes over time (that is, pre- to post-intervention). For each outcome measured, effect sizes (ES) will be computed overall and by site and compared to ES from the iHEAL trial to evaluate whether the benefits for women demonstrated in our research are maintained during implementation in community-based health care organizations.

As noted in the sample section above, and given our experience to date, we expect a final sample of ~224 women to complete these surveys. This is potentially large enough to also explore whether there are differences in the level of improvement for some groups of women based on their characteristics (e.g., those with and without heavy substance use, living with a partner versus separated), according to the type of organizational setting (Primary Health Care Center, Public Health Unit, Indigenous Wellness Center), or the delivery model used (e.g., whether nurses offer iHEAL only or concurrently with other programs). However, these decisions cannot be made a priori as the specific characteristics of women in the sample are unknown.

#### Integrated analysis.

The results of this study will draw on findings from both qualitative and quantitative analyses combined in an integrated way to address the research questions. We will use a number of strategies, including data matrices or visual displays, infographics, and case examples, to create findings that are ready to share with implementing organizations as part of the process of consolidating lessons and implications from this research through further discussion, reflection, and feedback.

## Ethical considerations

This study will be conducted in compliance with the Tri-Council Policy Statement: Ethical Conduct for Research Involving Humans (TCPS2) [36]. All members of the Research Team will complete mandatory ethics training (per organizational policy at each university).

### Informed consent

Organizations are formal partners in this study. The organization (rather than individual staff member) is the unit of analysis. Access to de-identified, group level program administrative data, and collection of high-level (anonymous) notes during meetings about iHEAL implementation are part of the agreement with each organization.

We will seek *individual* level informed consent from individual participants taking part in research activities as outlined in the methods section above. For all interviews, written or verbal consent will be established, either in person or via an electronic consent form, prior to participation. Where participation includes multiple activities, consent will be reaffirmed verbally before each interview/activity. All participants will receive a Letter of Information and will have opportunities to ask questions prior to beginning study activities.

### Voluntary participation

Participation in all research activities is completely voluntary. While organizations are formal partners in the study and have committed to participation in writing, individuals working at each organization are able to withdraw from individual interviews at any time. They may ask to have their interview data withdrawn from the study any time prior to analysis. Participation status of staff members will not be shared with the organization and will have no bearing on employment.

Participation in surveys and interviews for women accessing iHEAL is also completely voluntary. Women can refuse to answer questions or withdraw from surveys or interviews at any time. Participants may ask to have their data withdrawn from the study any time prior to analysis. Participation in research activities will not affect women’s enrollment in the iHEAL program, nor their access to supports and services.

### Safety

There are no known risks or harms for members of our partner organizations participating in this study. The risk of a privacy breach is small and will be mitigated by data being stored on a secure server at Western University behind the institutional firewall, with all study files password protected and only accessible to the study team.

Women participating in iHEAL may be living in the same household as an abusive partner or be living separately. Many live with risks to personal safety independent of the research context. It is possible that partners may become angry or abusive should they discover that women are taking part in this study. We expect that this risk may be higher for women who are living with abusive partners and/or who are unable to complete online surveys in a separate space.

Research staff will follow a safety protocol, developed and used with success in many previous studies, to guide all interactions with women [37]. To protect privacy and minimize the risk that a partner may discover a woman’s participation in this study, Research Coordinators will only contact each woman using the safe contact information she provides. Unless otherwise requested by the woman, her safe email address will be used as the default for all messages sent by the Research Team. In each message, women will be encouraged to contact the Research Team should their contact information change. Each participant will also be provided with online information on how to erase their browser history, and how to set their browser to incognito mode (so that history is not saved and cannot be discovered by others).

### Mild distress

Women who have experienced IPV may live with disturbing memories of their experiences for many years. However, as part of healing, over time, women find ways to manage the distress they feel, with some ‘thriving’ as they gain hope and a sense of control over their lives [38]. In this context, we expect that completing the questions in the online surveys or qualitative interviews may create mild distress for women as they reflect on their experiences of abuse. During completion of all research activities (surveys, interviews), women will be encouraged to take breaks as needed and will be provided with information about available services and how to access them. As previously noted, we will provide information about common signs of a stress reaction and strategies they can use to manage these reactions should they occur.

### Equity considerations

We will intentionally invite women from diverse social locations, backgrounds, and life experiences to participate in this research. This includes women who identify as Indigenous, newcomers, recent refugees, those living in rural and urban contexts, and of varied ages, gender identities, socioeconomic status, and family contexts, some of whom will have experienced discrimination, stigma, or other systemic barriers to health and well-being.

Research demonstrates that the higher incidences of violence experienced by Indigenous women is directly related to the legacy of colonization in Canada [39], yet responses to Indigenous women experiencing violence have been inadequate, despite national commissions calling for systems change and better direct care services [40]. Integrating guidance from Indigenous women with lived experience of IPV and knowledge from Indigenous Elders, iHEAL was adapted to address the needs of Indigenous women. Our Research Team has an opportunity to advance understandings about the organizational contexts and conditions that shape implementation of iHEAL in an urban setting with a mandate to serve Indigenous peoples, including Indigenous women who have experienced multiple forms of violence. Our team acknowledges the history of exploitation of Indigenous peoples in research, and we have sought to reflect this awareness in designing this study. Specifically, iHEAL research staff have all completed the San’yas Indigenous Cultural Safety Program [24] to ensure all aspects of the research are grounded in culturally safe and antiracist approaches.

To promote safe participation of women with many different life experiences, including Indigenous women, we are using a continuous process of inquiry and confirmation of consent. Our ways of involving women in research are inherently strengths-based, woman-led, trauma-and violence-informed, and informed by cultural safety. Our procedures include attention to cultural and emotional safety as well as physical safety. In this vein, the surveys and open-ended interview questions to be used have been reviewed by Implementation Team members who work with women from many different backgrounds and have been adjusted accordingly.

## Conclusion

This study protocol outlines a mixed method, multi-site research plan to examine the implementation of iHEAL across 3 diverse community-based health care organizations in 3 provinces in Canada. Our aim is to generate new knowledge about the complexities and challenges inherent in delivering iHEAL, including the organizational resources and supports required to enable nurses and other team members to implement the program effectively and with fidelity. By integrating quantitative and qualitative data, the research will provide a comprehensive understanding of implementation processes for iHEAL in these organizations and the structures and supports necessary at an organizational level to sustain the implementation and delivery of iHEAL in real-world settings.
